# Mobilization of Unexploded Ordnance on the Seabed

**DOI:** 10.3390/toxics10070389

**Published:** 2022-07-13

**Authors:** Peter Menzel, Anja Drews, Tjark Mehring, Christoph Otto, Dorthe Reng Erbs-Hansen

**Affiliations:** 1 Corvus Works UG, Ernst-Rieck-Str. 6, 18225 Kühlungsborn, Germany; 2 TenneT, Eisenbahnlängsweg 2a, 31275 Lehrte, Germany; anja.drews@tennet.eu (A.D.); tjark.mehring@tennet.eu (T.M.); 3 Chair of Ocean Engineering, University of Rostock, Albert-Einstein-Str. 2, 18059 Rostock, Germany; christoph.otto@uni-rostock.de; 4 Vattenfall Vindkraft A/S, Jupitervej 6, 6000 Kolding, Denmark; dorthe@erbs-hansen.dk

**Keywords:** UXO, burial, sediment transport, mobilization, Morrison equation, hydrodynamics

## Abstract

Unexploded ordnance devices (UXO) pose a potential threat to human life and material during offshore construction activities. Extensive survey activities are conducted to locate, identify, and clear these objects as necessary. For the period thereafter, it is necessary to investigate whether areas that have already been cleared, or even objects that remain in place, may be affected by mobilization under tidal currents or waves, and could thus have an impact on operation and maintenance during the lifetime of the offshore installation. In this study, model simulations based on fluid mechanics are described to derive the loads on the objects caused by currents and waves and combined with knowledge of the known burial condition of the objects. Within the model, the hydrodynamic and hydrostatic loads on the object caused by waves and currents are balanced with inertia and rolling resistance. Thus, the critical current velocity and critical wave conditions for the mobilization of different objects are calculated and compared with the environmental conditions prevailing in the North Sea. As a result, a recurrence interval for the potential mobilization of objects on the seafloor is given, which can now be used to optimize route surveys and thus help accelerate offshore construction work. It is shown that currents are not able to mobilize the objects investigated in the study in almost all regions of the North Sea. Waves can mobilize certain objects in very shallow and extreme conditions.

## 1. Introduction

As a result of military conflicts and exercises, unknown quantities of unexploded ordnance (UXO) lie on the seabed in numerous marine regions of the world. According to [1], about 1.3 million tons of unexploded ordnance are estimated in the German Bight (North Sea) alone. This UXO can pose a threat to the lives and equipment of maritime users. In particular, during offshore construction work that encroaches on the seabed, such as the installation of offshore wind turbine foundations or cables, a safe working environment must be created by clearing unexploded ordnance. The energy transition in many European countries requires accelerating the installation of complete offshore infrastructures, so flexibility is needed in planning UXO clearance work prior to installation. To assess the probability of recontamination of an already cleared region, a better understanding of the processes and time scales for the mobilization of objects on the seabed is needed. In [2], a probabilistic model for the mobilization of small UXO was presented and successfully tested. However, the applicability of this model to large objects in the North Sea is severely limited. Therefore, the critical current velocity and critical wave condition are modeled for two different types of UXO common in the North Sea region. The model is applied to various offshore developments in the North Sea. The model for flow-induced mobilization was verified by laboratory experiments presented in [3]. The model for wave-induced mobilization is based on theoretical calculations. Determining the factors affecting the mobilization of unexploded ordnance on the seafloor and objects buried in the seafloor will allow offshore developers to evaluate the usefulness of previously recorded survey data and clearance work to keep the area safe for work.

**Figure 1:**
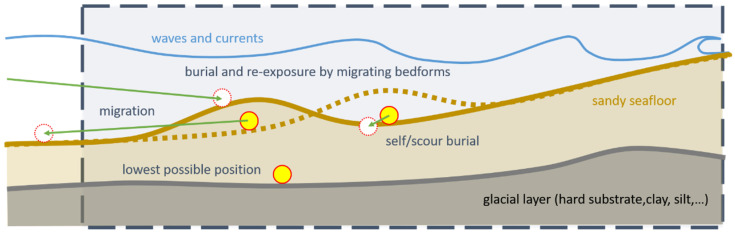
Conceptual drawing of possible processes of burial, re-exposure, and migration of UXO in the area of interest. Yellow circles with red outline represent the original position of the UXO, white circles with dashed red outline represent the position after migration, with the direction indicated by a green arrow.

Figure 1 shows the processes that can occur in an area of interest. In an area with a mobile sediment layer covering a hard clay or silt layer, the sediment layer can form various bed forms such as ripples, mega-ripples, sandbars, and sand waves. Most of these forms are caused by hydrodynamics (waves and currents).

## 2. Methods

### 2.1. Burial of Objects

To analyze the mobilization of objects on the seabed, the state of their burial must be known. This is basic information. Burial of objects is caused by various effects. Numerous research works deal with the subsequent burial depths due to self-burial, especially for cylindrical objects [2,4,5]. Self-burial is caused by the flow structure around the object.

**Figure 2:**
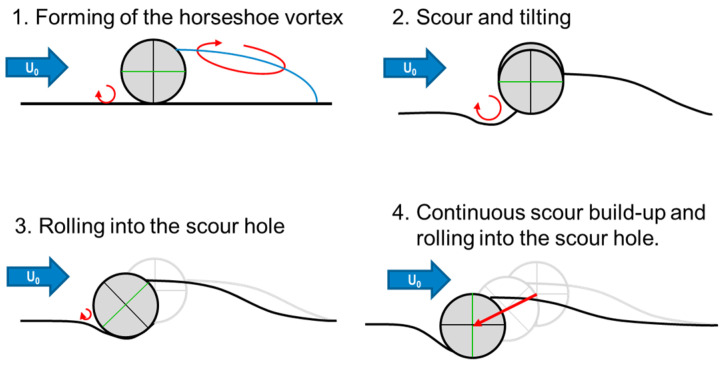
Model of burial of a cylindrical object in constant inflow [6]. Incident current from the left side.

The process of self-burial due to currents shown in Figure 2 was published in [6,7]. As shown in [8], the preferred orientation of a cylindrical object on the seafloor is perpendicular to the incident current, caused by the Munk moment described in [9]. Due to the presence of the object, the bottom boundary layer in front of the object forms a vortex—the horseshoe vortex. This vortex leads to a very thin bottom boundary layer, which results in a high load on the sediment particles eroding in this area. The resulting trough becomes larger and larger until the object begins to move into it. An arcuate vortex in the wake forms the recirculation area, which acts as a sorting sediment trap. The formed region of accumulation prevents the object from rolling downstream. The upstream and downward displacement of the object occurs until the object is buried by about 115% of its diameter, as published in [4]. Thus, the horizontal displacement is limited to about one diameter of the object. Compared to the goal of this study, to determine if an object can be mobilized to travel over longer distances, this is a small displacement. At this point, it is important to note that the displacement caused by self-burial must be distinguished from object mobilization, which can enable migration over greater distances.

Another process of object burial is caused by far-field effects that occur independently of the object in place. These effects are general seafloor changes and large-scale ripples (mega-ripples) that can be observed in highly morphodynamic regions such as the North Sea or estuaries. In these effects, the object is not mobilized, but sediment is deposited on the object. These effects can also lead to re-exposure of the objects and must therefore be taken into account when analyzing the mobilization of objects.

The effects described above occur on sandy seabeds. In areas with mud, objects can sink into the mud without external forces acting on the object or the sediment. In contrast, objects located on silt or clay are not buried because this sediment is usually not eroded by currents and waves. Similar behavior is observed on rocky substrates and in areas with high seagrass beds. Therefore, these regions must be treated separately.

### 2.2. Current-Induced Mobilization

Assuming that an object on a sandy seabed is at least partially but not completely buried by self-burial, it is still subject to flow- and wave-induced stresses. In [3], wind tunnel experiments, experiments in large flow tanks, and numerical simulations were used to determine the critical flow velocity, $U_{crit}$, required to mobilize an object from a partially buried position.

(1) $$U_{crit}=\sqrt[{b}]{\frac{\rho_{w}gD_{avg}V\left(\rho_{obj}-\rho_{w}\right)}{2a\mu^{2}L}}\cdot \frac{\mu }{\rho_{w}\left(1-z_{b}^{*}\right)D_{avg}}.$$

This velocity is defined to attack 20 cm above the seabed. The parameters in this equation are the density of the water $\rho_{w}$ and of the object $\rho_{obj}$, the gravitational acceleration g, the dynamic viscosity μ, the average diameter of the object $D_{avg}$, its length L, and its volume $V_{obj}$. The burial depth $z_{b}^{*}$ describes the fraction of the object diameter that is buried. The parameters a=0.2315 and b=2.1137 are determined by statistics and also published in [3].

### 2.3. Wave-Induced Mobilization

Especially in shallow waters, the influence of surface waves on objects on the seafloor cannot be neglected. Compared to the wavelength of the surface wave, it can be assumed that large parts of the North Sea are shallow during a considerable number of days per year. More precisely, the hydrodynamic effects of surface waves must be considered during most of the year for almost the entire German Bight. This changes in the protected areas of the Wadden Sea, where the wave effect can be neglected.

**Figure 3:**
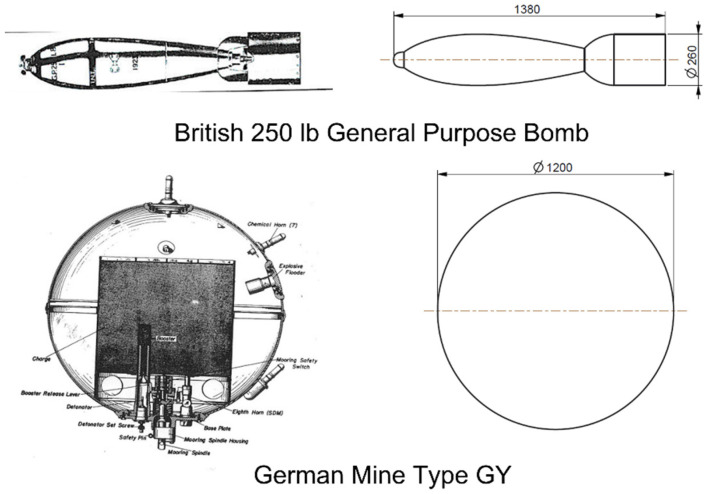
Drawings of the axially symmetric objects and their simplified models [10].

Quantifying the critical surface waves that trigger the mobilization of an object on the seafloor is much more complex than quantifying the critical current velocity because many more unknown variables must be considered. The analysis published in [10] is applied to typical UXO found in the North Sea. These are, for example, the British 250-pound general purpose bomb and the German mine type GY, as shown in Figure 3. The German mine type GY is assumed to be completely flooded. The absolute mass of the British 250-pound general purpose bomb is given as m=112 kg, while the flooded absolute mass of the German mine type GY is approximated by m=1386 kg. All dimensions are given in Table 1.

**Figure 4:**
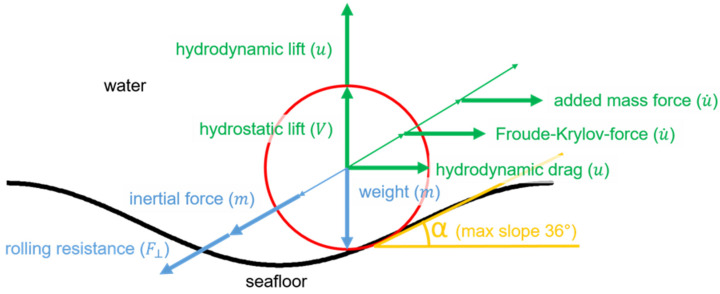
Loads on a cylindrical object in a symmetrical scour cavity in the presence of waves [10].

In contrast to flow-dominated scenarios, the scour trough shown in Figure 2 builds up on both sides of the object, such that the accumulation area in the “wake” shown in [7] is absent. Under this assumption, the load balancing model with a sinusoidal scour trough published in [10] and shown in Figure 4 can be applied.

Using the relationship published in [11], known as the Morrison equation, the horizontal loads can be balanced as follows:

(2) $$1.5\cdot m{\dot{u}}_{obj}=\rho V_{obj}\dot{u}+\rho c_{a}V_{obj}\left(\dot{u}-{\dot{u}}_{obj}\right)+\frac{\rho }{2}\left(u-u_{obj}\right)\left|u-u_{obj}\right|c_{d}A_{obj}$$

where m is the mass of the object; $V_{obj}$ and $A_{obj}$ are the volume and cross-section of the object, respectively; u and $\dot{u}$ are the horizontal velocity and acceleration of the water, respectively; $u_{obj}$ and ${\dot{u}}_{obj}$ are the horizontal velocity and acceleration of the object, respectively; ρ is the density of the water; $c_{a}$ is the coefficient of added mass, and $c_{d}$ is the drag coefficient. On the left side, the inertia is determined taking into account the rotation. Since the mass distribution of the objects is not completely known, a homogeneous mass distribution is assumed. On the right side of the equation, we find the Froude–Krylov force, the added mass force, and the hydrodynamic drag force. The hydrodynamic lift force is considered for the vertical component. The governing coefficients in this equation are the dimensionless coefficients for drag, lift, and added mass, which are not constant with the Reynolds number and burial depth. For the objects considered here, they are about $0.2<c_{d}<0.7$ and $0.2<c_{l}<0.7$.

The dimensionless coefficients in Equation (2) were derived from experiments and CFD simulations for the objects in Figure 3. They depend on several variables such as the Reynolds number and burial depth. The velocity and acceleration of the water at the location of the objects were derived from the wave period, wave height, and water depth using Stokes’ wave theory of 3rd order. By substituting all of the above variables into Equation (2) and the equivalent equation for the vertical loads, the acceleration of the object can be calculated.

### 2.4. Critical Waves for Mobilization

**Figure 5:**
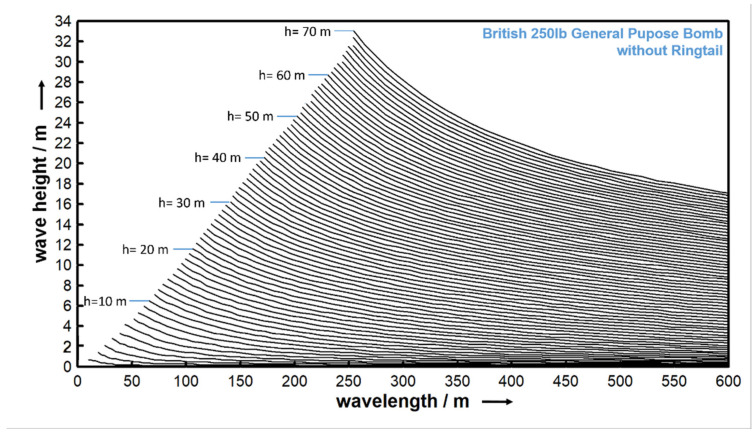
Critical conditions for the British 250-pound general purpose bomb without a ring tail. In wave conditions above or right of the graph, objects are mobilized; left and below, they do not get mobilized.

The wave-induced mobilization of objects can be derived from the equations presented above. In practice, the equation for wave-induced mobilization cannot be solved directly. Therefore, a simulation method must be used. A numerical integration of these equations over time yields a motion simulation that is used to decide whether the object leaves the scour trough and is thus mobilized, or whether it remains within the trough. For this simulation, 10 monochromatic waves were considered and resolved by 360 phase steps each. By testing different water depths (1 m≤h≤70 m with 0.1 m discretization), wavelengths, and periods (1 s≤T≤10 s with 0.1 s discretization), the critical parameter combinations leading to mobilization of a single object at a single burial depth were determined. The results of this simulation for the British 250-pound general purpose bomb, neglecting the ring tail, are exemplarily shown in Figure 5.

Each curve in the diagram represents the critical wave height for a given water depth at a burial depth of 50% of the object diameter ($z_{b}/D=0.5$). Higher wave heights and longer wavelengths mobilize the object. However, these generalized results can later be related to wave data for a particular site, whereas data from a site (e.g., from weather forecasts or directly measured) are usually available as statistical data that must first be converted to individual waves. As a worst-case scenario, the maximum wave height and peak wave period were used for further analysis.

### 2.5. Recurrence Interval

The results shown in Figure 5 can be used for monitoring purposes by comparing them, for example, with short-term weather forecasts or real-time wave monitoring data. However, for planning purposes and to assess the likelihood of recurrent mobilization in a given sea area, the analysis of historical data from a spatial wave model, as performed in this study, can be very useful. Here, the recurrence interval (or recurrence period) method is a common method for statistically predicting the time between special or extreme events from historical observations. Typically, this method is used for severe storm events or similar extreme weather situations that typically occur every 10, 20, 50, or 100 years. For these analyses, the recurrence time $t_{r}$ is derived by hand from a time series of m events within n years or days as follows:

(3) $$t_{r}=\frac{n+1}{m}\;$$

However, determining the average recurrence interval of the mobilization of objects on the seafloor is a complex matter. This is primarily because these events tend to occur much more frequently at certain times of the year, such as during the storm season. For example, if an object can be mobilized once per day for two consecutive months during the local storm season, determining the return times using the above approach would yield a return time of about 366/60 = 6.1 days. However, the desired result would be more of a return time of one year, in the sense that it migrates once a year on a consecutive number of days, so that wind farm operators know that checking mobilization once a year is sufficient.

The analysis of these historical data usually requires the evaluation of both temporally and spatially large datasets. In order to be able to automatically evaluate the mobilization return times with the desired result mentioned above, a new automatic algorithm was developed in this research. In the first step, the object mobilization is determined on a daily basis with the binary information “mobilization” or “no mobilization” on a spatial and temporal grid of arbitrary resolution. This is necessary because wave-induced mobilization depends strongly on local conditions such as water depth, which changes rapidly near the coast; local flow systems near river mouths, and similar effects. In addition, seafloor sediments and seafloor mobility must be considered. Therefore, high spatial resolution for the recurrence interval is required for decision making.

**Figure 6:**
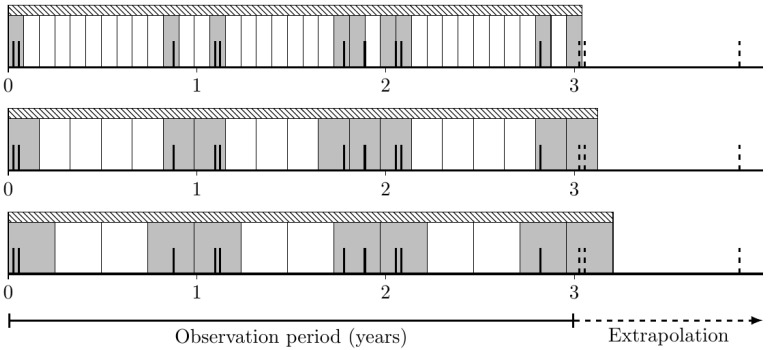
Method of different interval lengths, with events shown as bold black vertical bars. Gray intervals contain events and white intervals contain no events. The shaded bar shows the time scale analyzed.

As mentioned earlier, mobilization events accumulate during certain periods of the year. This accumulation is observed because, for example, a single storm event may last several days, resulting in repeated mobilization of an object during that time. If only Equation (3) were used, this would result in multiple counts of an event that is actually a single event. To solve this problem, the entire observation period is divided into a different number of time intervals. This is performed several times with different interval lengths, where the shortest length is one day and the longest is half of the total observation period. The principle is illustrated in Figure 6. For each interval length, the number of intervals containing an event is counted (see gray intervals in Figure 6).

It should be noted that the total observation period is usually not an integer multiple of the interval length under investigation. This remainder becomes more significant the longer the interval length is. To include the information in the remainder, the observed data must be extrapolated by the missing information at the end to fill the last interval. For this purpose, the data from the beginning of the observation period are simply appended to the end of the data series.

The recurrence interval is then determined from the proportion of intervals that contain an event. For this purpose, the interval length is determined where every second interval contains an event. The corresponding average recurrence time is then to be determined as twice the interval length, since there is then, on average, an interval that does not contain an event between two intervals that do contain an event. Accordingly, the time from the center of an interval containing an event to the center of the next corresponding interval is just twice the interval length. An example of this procedure is shown in Figure 6, where the last interval length satisfies this criterion and yields a return time of about half a year. Although it can be argued that a return time of one year might be even more realistic, this approach produces more realistic results than applying Equation (3) but is still conservative.

**Figure 7:**
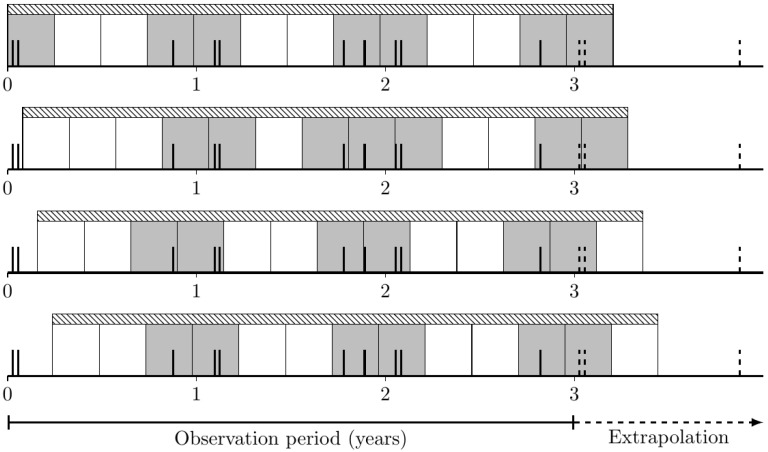
Shifted intervals to minimize the impact of events near the interval boundaries. Events are shown as bold black vertical bars. Gray intervals contain events and white intervals contain no events. The shaded bar shows the time scale analyzed.

Finally, several events may occur close to the edge of the intervals containing them, leading to significantly different results if the entire observation frame is shifted by only a small number of days. To minimize the impact of these effects of events at the edge of intervals, the analysis is repeated several times for each interval length by shifting the beginning of the first interval upward by all possible days into the corresponding interval length to be analyzed, as shown in Figure 7. The number of intervals with events is then averaged for all analyses with the same interval length.

### 2.6. Obtaining Regional Information through Spatial Planning

For spatial planning, it may be necessary to perform a recurrence interval analysis for the mobilization of a particular object of interest in a region of interest. From Equations (1) and (2), it can be seen that the geometric parameters of the object, water depth, current, and wave information are needed.

The composition of the seafloor (sediment types) was taken from the “Geopotenzial Deutsche Nordsee (gpdn)-project”, described in [12,13]. The near-bottom tidal currents were taken from the BSH Operational Circulation Model (BSHcmod). The wave parameters were taken from the German Weather Service (DWD) and its Coastal Wave Model (CWAM) and European Wave Model (EWAM). The data used represent the forecasts for the next 12 h until the next model run in 3-hour increments. A complete list of all input data can be found in Table 2. The far-field accumulation and erosion data are from the AufMod project, published in [14]. These data were used to determine the object-specific mobilization characteristics.

**Figure 8:**
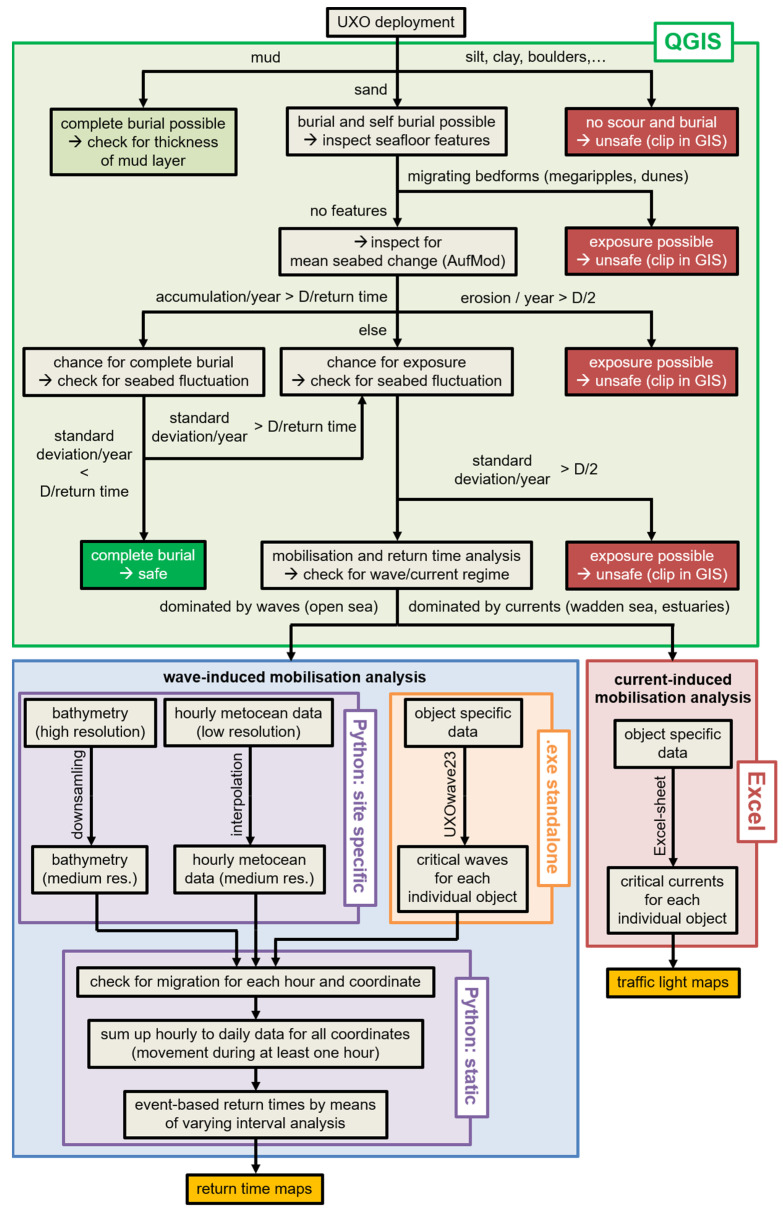
Steps of data processing.

The spatial information, including the maps of the recurrence interval for a given object, is processed in QGIS based on a hierarchical decision tree shown in Figure 8, which does not necessarily represent a schedule of processing but shows the important decisions. In practice, both wave-induced mobilization analysis and flow-induced mobilization analysis run separately and are used as inputs to the GIS.

However, the first GIS analysis is based on sediment type. Here, it is assumed that an object located in a muddy area may be immobile for a long time due to the heavy burial and suction effect, while objects on hard substrates such as silt and clay may not even be buried and therefore be very mobile. Since the physical processes in these areas are not considered, these regions are excluded from further analysis (cut away in the GIS).

For some regions, seafloor morphology datasets are available from an extended seafloor analysis. These datasets include regions where mega-ripples and dunes are present. Within these regions, further analysis can be performed depending on the quality of the information. If only the presence of mega-ripples is indicated, it is assumed that objects may be completely buried but may also be uncovered after an unknown time. Therefore, these regions are also excluded from the GIS analysis as long as no further information is available.

**Figure 9:**
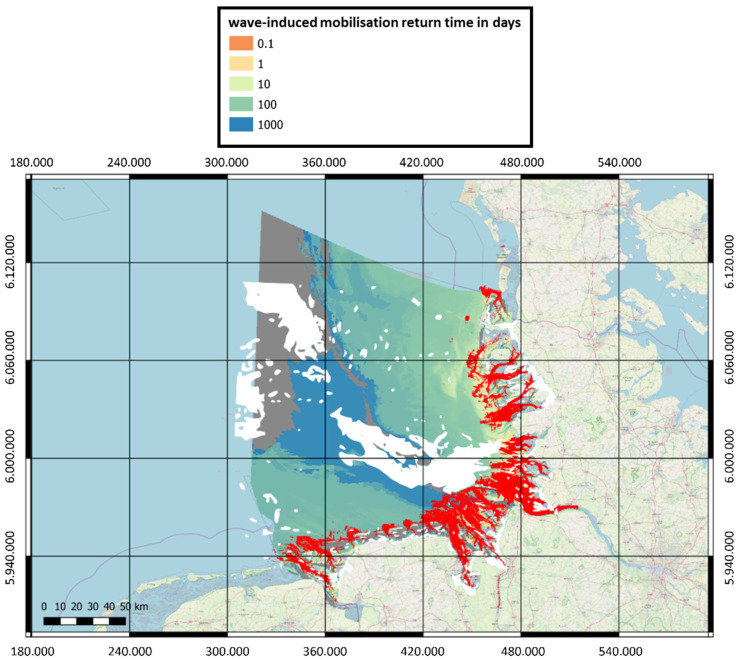
Return period map for wave-induced mobilization of the British 250-pound General Purpose Bomb in EPSG:25832-ETRS89/UTM Zone 32 N. The red areas represent regions of sediment erosion greater than D/2 for this object.

Further analysis of seafloor morphology will be performed using information from the AufMod project. From these data, the mean annual change in the seafloor and the annual standard deviation are calculated. A distinction is made between mean annual accumulation of more than half an object diameter, erosion of more than half an object diameter, and change of less than half an object diameter. Depending on which case occurs, complete exposure or the chance of complete burial is assumed. In the case of severe sediment erosion, the object may be fully exposed. Unless the self-burial process is described by a model, it is assumed that the object can be mobilized very easily. A recurrence interval for mobilization cannot be specified for this case. For the alternative cases, another analysis is performed considering the standard deviation of the annual seafloor change. If the mobilization return time is large compared to the annual standard deviation of seafloor change, the standard deviation can be neglected and the object is assumed to be completely buried. Otherwise, its value must be checked, which is done even in the case of low mean accumulation and erosion. If the annual standard deviation is greater than half of the object diameter, the object may be completely exposed from time to time and thus be highly mobile. Regions with this behavior are considered unsafe and therefore excluded from the map of return periods in the GIS. In the case of a low standard deviation, the return time method and mobilization prediction are applicable. These are the remaining regions in the map shown in Figure 9.

## 3. Results

The main result of the above analysis comprises maps of mobilization return time for the objects of interest. Figure 9 shows an example of the mobilization time for the British 250-pound general purpose bomb. The white areas represent the regions that were excluded from further analysis according to the path described above and in Figure 8. The gray areas represent the regions whose recurrence intervals are greater than the observation period of about four years.

**Figure 10:**
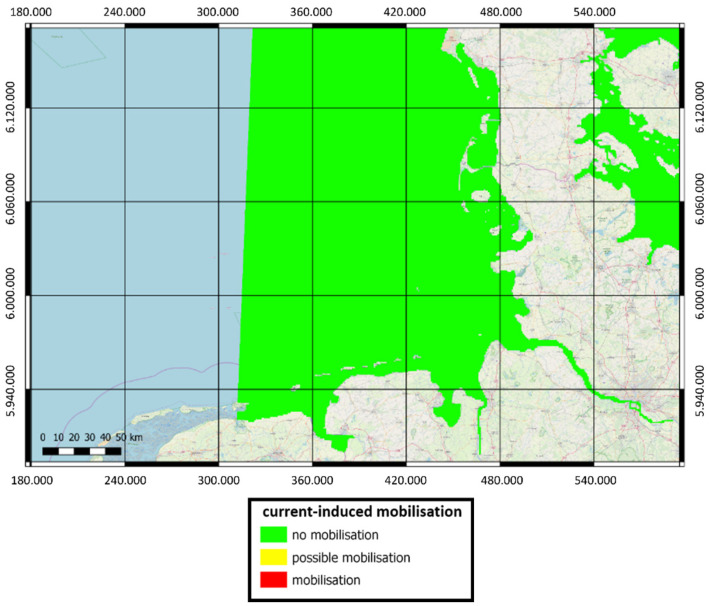
Regions of current-induced mobilization for the British 250-pound general purpose bomb and the German mine type GY in EPSG: 25832-ETRS89/UTM Zone 32 N.

Current-induced mobilization is accounted for by a separate attribute since it occurs only in very strong tidal channels and thus depends on the tides rather than the wave scenario. The German mine type GY and the British 250-pound general purpose bomb are not mobilized at all in the entire German Bight, as can be seen in Figure 10.

**Figure 11:**
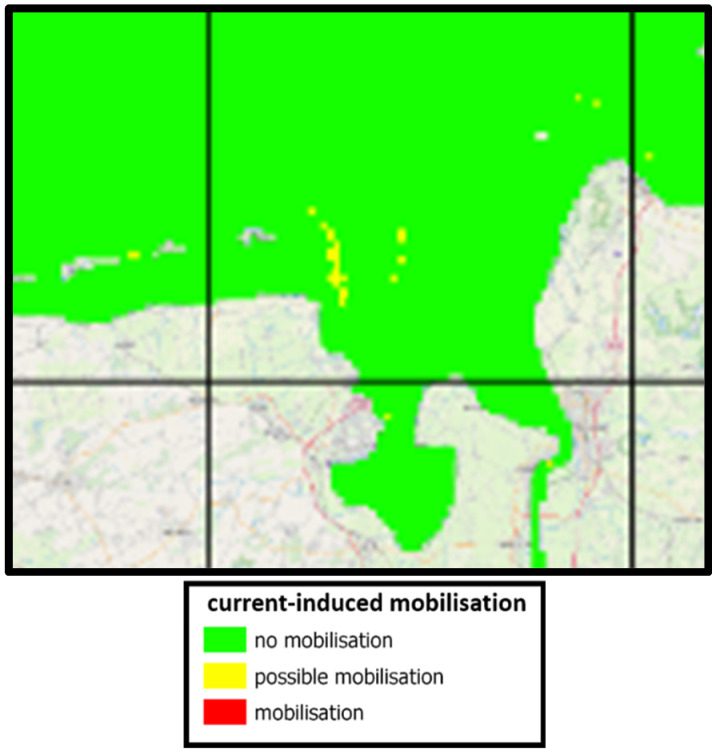
Regions of current-induced mobilization for a small object showing possible mobilization in EPSG: 25832-ETRS89/UTM zone 32 N.

In addition to the German mine type GY and the British 250-pound general purpose bomb, a number of other objects were investigated that show very similar results with respect to flow- and wave-induced mobilization. In particular, flow-induced mobilization does not occur for all objects. Only for very small and light objects can some mobilization potential be detected in the jade region, as shown in Figure 11.

## 4. Discussion

The results of this research are based on a spatial historical analysis of DWD wave forecast data. These data include spatial wave data that account for water depth, tides, wind forcing, sea state, and attenuation effects. Thus, extreme events are also included in the dataset. The uncertainty of these data is not directly reported because these forecast simulations depend on many external factors. The wave model export data are statistical data that result in a significant wave height $H_{s}$ and a peak wave period $T_{p}$. To compare these results with the results of the mobilization model, which uses monochromatic, unidirectional waves, the statistical data must be converted to individual waves. In the analysis, the maximum wave height $H_{max}$ and $T_{p}$ are used. Since the two do not normally occur simultaneously and are usually the result of overlapping multidirectional waves, the results are assumed to represent worst-case scenarios. Continuing this article, realistic wave spectra and multidirectional waves could improve the results.

The ability of hydrodynamic forces to mobilize an object on the seafloor depends strongly on the burial state of the object. The processes of burial and re-exposure of objects on the seafloor are explicitly not the subject of this article. Nevertheless, some assumptions about the geometry of scour from cited articles are used. A combination of burial and mobilization could be of great interest for future research, as well as validation of the results with field data. This could be done by extensive field tests of very long duration and complex measurement equipment. Flume tank tests can be performed to validate individual processes described by the model. For flow-induced mobilization, this was conducted with real-scale objects by [3]. For wave-induced mobilization, no full-scale flume tank tests have yet been published for large objects.

Since the recurrence interval is a statistical analysis, the results cannot be directly applied to the future as the time between real events. The probability that two events follow each other earlier than or at the specified recurrence interval is 50%, as is the probability that two events follow each other later than or at the recurrence interval.

## 5. Conclusions

The self-burial processes of objects on the seafloor are well known based on many years of research and form the basis for research on the mobilization of UXO. A distinction is made between small displacements of objects on the seafloor as a result of self-burial and mobilization of an object that displaces the object from its position and can lead to long-distance migration. The instruments described above can be used to predict the critical conditions for current- and wave-induced mobilization of unexploded ordnance on a sandy seafloor. If weather conditions are known from measurements or models, the results can be used to quantify whether an object can be mobilized by waves or currents. This information can be used for monitoring purposes and predictive spatial analysis. An automated method for calculating the recurrence interval of potential mobilization of objects on the seafloor has been described. This method accounts for clustered and seasonal events, providing more useful results than the typical simple approach. The analysis considers bathymetry, seafloor sediments, seafloor morphology, and seafloor features using a hierarchical decision tree and expert knowledge.

## Figures and Tables

**Table 1:** Dimensions of the objects.

Object	Length (cm)	Diameter (cm)	Mass (kg)	Volume (L)	Density (kg/m^3^)
250 lb Bomb	97	26	112	33	3394
Mine GY	120	120	1386	905	1531

**Table 2:** List of input data used.

Data	Format	Resolution	Source
UXO Object-specific data			
Object-specific data; dimensions, weight	Excel	-	Provided by the project owner
**Mobilization data**			
Object-specific mobilization data	TXT	Water depth: 1 m Wave height: 0.1 m Wave period: 0.25 s	UXOwave23 is based on object-specific data
**Bathymetry and hydrology**			
Bathymetry	GeoTiff	50 m gridded data	Federal Maritime and Hydrographic Agency (BSH); Vertical reference: Lowest astronomical tide
Change in bathymetry	TXT	50 m gridded data 1982–2012 ∆T = 1 year	AufMod project
Wave data	netCDF	900 m gridded data 10/2016–09/2019 ∆T = 3 h	German Weather Service (DWD)
Current data	netCDF	Lat.: 30 s Longer: 50 s Depth: 5 m 01/2008–12/2018	Federal Maritime and Hydrographic Agency in Germany (BSH) KU-Grid of the BSHcmod, NOKU facility V4

## Data Availability

No further data available for publication.

## References

[1] Böttcher C., Knobloch T., Rühl N.-P., Sternheim J., Wichert U., Wöhler J. (2011). Munitionsbelastung der Deutschen Meeresgewässer—Bestandsaufnahme und Empfehlungen.

[2] Rennie S.E., Brandt A., Friedrichs C.T. (2017). Initiation of motion and scour burial of objects underwater. Ocean Eng..

[3] Menzel P., Schütt C., Wranik H., Paschen M., Drews A. (2018). Towards a general prediction model for the current-induced mobilization of objects on the sea floor. Ocean. Eng..

[4] Whitehouse R. (1998). Scour at Marine Structures—A Manual for Practical Applications.

[5] Inman D.L., Jenkins S.A. (1996). A Chronology of Ground Mine Studies and Scour Modeling in the Vicinity of La Jolla ser.

[6] Menzel P., Leder A., Czarske J., Büttner L., Fischer A., Ruck B., Leder A., Dopheide Versandung eines Zylinderabschnitts unter Einfluss von Oberflächenwellen im Laborversuch. Proceedings of the Proceedings der 23. GALA-Fachtagung “Lasermethoden in der Strömungsmesstechnik“.

[7] Menzel P., Leder A., Kähler C., Hain R., Cierpka C., Ruck B., Leder A., Dopheide D. (2013). Kolkbildung im Umfeld zylindrischer Objekte sowie deren Versandung im Wasserkanalexperiment. Proceedings of the Proceedings der 21. GALA-Fachtagung “Lasermethoden in der Strömungsmesstechnik“.

[8] Menzel P., Witte M., Leder A., Leder A., Brede M., Ruck B., Dopheide D. (2012). Windkanalexperimente zur Bestimmung der Strömungsstrukturen um einen quer zur Anströmung auf einer Bodenplatte lagernden Zylinderabschnitt. Proceedings of the Proceedings der 20. GALA-Fachtagung “Lasermethoden in der Strömungsmesstechnik“.

[9] Munk W.H. (1944). Proposed Uniform Procedure for Observing Waves and Interpreting Instrument Records.

[10] Menzel P., Drews A., Otto C., Schütt C., Papadakis J.S. Mobilization of UXO, caused by hydrodynamics. Proceedings of the 5th Underwater Acoustic Conference and Exhibition UACE 2019.

[11] Morison J.R., O’Brien M.P., Johnson S.A., Schaaf S.A. (1950). The force exerted by surface waves on piles. Pet. Trans. AIME.

[12] Figge K. (1981). Sedimentverteilung in der Deutschen Bucht (Blatt: 2900, Maßstab: 1:250.000).

[13] (2003). Geotechnische Erkundung und Untersuchung—Benennung, Beschreibung und Klassifizierung von Böden—Teil 1: Benennung und Beschreibung.

[14] Die Küste (2015). Aufbau von integrierten Modellsystemen zur Analyse der langfristigen Morphodynamik in der Deutschen Bucht AufMod.

